# Nitrogen and sulfur-doped carbon quantum dots as fluorescent nanoprobes for spectrofluorimetric determination of olanzapine and diazepam in biological fluids and dosage forms: application to content uniformity testing

**DOI:** 10.1186/s13065-022-00894-y

**Published:** 2022-11-15

**Authors:** Galal Magdy, Noura Said, Ramadan A. El-Domany, Fathalla Belal

**Affiliations:** 1 Pharmaceutical Analytical Chemistry Department, Faculty of Pharmacy, Kafrelsheikh University, P.O. Box 33511, Kafrelsheikh, Egypt; 2 Microbiology and Immunology Department, Faculty of Pharmacy, Kafrelsheikh University, P.O. Box 33511, Kafrelsheikh, Egypt; 3 Pharmaceutical Analytical Chemistry Department, Faculty of Pharmacy, Mansoura University, P.O. Box 35516, Mansoura, Egypt

**Keywords:** Nitrogen, sulfur-doped carbon quantum dots, Olanzapine, Diazepam, Fluorescence quenching, Content uniformity

## Abstract

A validated, sensitive, and simple spectrofluorimetric method was developed for the analysis of two important CNS-acting drugs, olanzapine and diazepam, in their commercial tablets without the need for any pretreatment steps. The developed method relied on the quantitative quenching effect of each of olanzapine and diazepam on the native fluorescence of nitrogen and sulfur-doped carbon quantum dots (NS@CQDs). NS@CQDs were prepared from thiosemicarbazide and citric acid by a facile one-pot hydrothermal technique. The synthesized NS@CQDs were characterized by different spectroscopic and microscopic techniques. NS@CQDs produced a maximum emission peak at 430 nm using 360 nm as an excitation wavelength. Calibration curves showed a good linear regression over the range of 5.0–200.0 and 1.0–100.0 μM with detection limits of 0.68 and 0.29 μM for olanzapine and diazepam, respectively. The adopted method was used for the determination of the investigated drugs in their tablets with high % recoveries (98.84–101.70%) and low % RSD values (< 2%). As diazepam is one of the most commonly abused benzodiazepines, the developed method was successfully applied for its determination in spiked human plasma with high % recoveries and low % RSD values, providing further insights for monitoring its potential abuse. The quenching mechanism was also studied and confirmed to be through dynamic and static quenching for olanzapine and diazepam, respectively. Due to the high selectivity and sensitivity, content uniformity testing of low-dose tablets was successfully performed by applying the United States Pharmacopoeia guidelines. The method's validation was performed in compliance with ICHQ2 (R1) recommendations.

**Supplementary Information:**

The online version contains supplementary material available at 10.1186/s13065-022-00894-y.

## Introduction

**Fig. 1:**
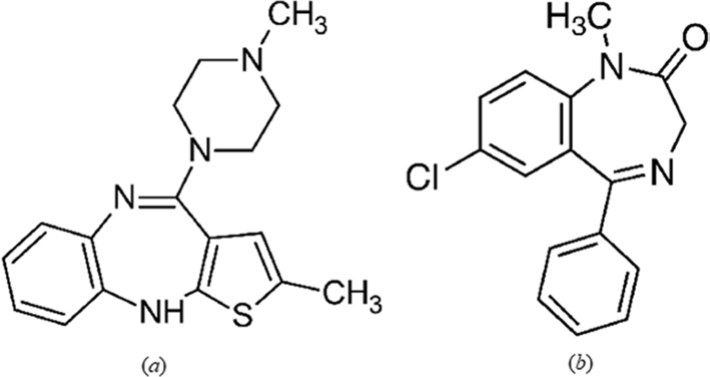
Chemical structure of OLZ (**a**), DZP (**b**)

Olanzapine (OLZ) is an atypical antipsychotic medication, which is used for the management of schizophrenia [1]. It is prescribed for the treatment and prevention of manic episodes and is used in the treatment of depression disorders when combined with fluoxetine. It has antagonistic effects on adrenergic, serotonin, and dopaminergic receptors, which is a characteristic property of most antipsychotics [2]. Its higher affinity for 5-hydroxytryptamine receptors over dopamine 2 receptors reduces the extrapyramidal effects such as tardive dyskinesia [3]. Chemically, OLZ is 2-methyl-4-(4-methylpiperazin-1-yl)-10H-thieno[2,3-b][1,5] benzodiazepine [4] (Fig. 1a).

Diazepam (DZP) is a benzodiazepine anxiolytic used for the treatment of anxiety disorders, severe muscle spasms, insomnia, panic attacks, and acute recurrent convulsive seizures [5]. Sedation in intensive care units (ICU) and short-term treatment of spasticity associated with neurologic disorders in children are two off-label uses of DZP [6]. It is also one of the most commonly abused benzodiazepines by young drug users, often in large doses leading to both psychological and physical dependence. Hence, quality control of benzodiazepines is critical, and specimen measurements are common [7]. The proposed method could be applied for the determination of high concentrations of DZP in spiked plasma samples, providing a method for identification of the drug abuse in forensic and toxicological analysis. Chemically, DZP is 7-chloro-1-methyl-5-phenyl-3H-1,4-benzodiazepin-2-one [8] (Fig. 1b).

There is a clinical evidence that combining antipsychotics and benzodiazepines results in a better treatment outcome in schizophrenia in terms of both positive and negative symptoms [9]. Acute psychosis is a rapid deterioration of a person's mental state, leading to agitated or aggressive behavior that may be dangerous to the person with psychosis, so benzodiazepines can be taken alone or in conjunction with antipsychotics to avoid this harm [10].

Several reports have been published for the assay of OLZ, such as LC–MS [11, 12], HPLC [13], GC [14], electrochemical methods [15, 16], spectrophotometry [17, 18], and spectrofluorimetry [19, 20]. Similarly, several methods for the determination of DZP have been reported, such as LC–MS [21, 22], HPLC [23], GC [24], electrochemical methods [25], spectrophotometry [26, 27], and spectrofluorimetry [28].

In this study, the suggested spectrofluorimetric method provides an easy, rapid, and cost-effective analytical solution for the analysis of each OLZ or DZP in their pharmaceutical preparations compared to the reported methods that need tedious pretreatment steps [19] and high-cost instrumentation [21–24]. Moreover, content uniformity testing was also conducted by adopting the United States Pharmacopoeia (USP) guidelines to produce high accuracy of the developed method [29]. Content uniformity is one of the most important tests that evaluate the batch's quality in a therapeutic product specification [30–32]. Testing of content uniformity ensures that the patient receives the correct dose and confirms that the strength of a drug remains in the specified acceptance values [31]. The proposed method depends on using nitrogen and sulfur-doped carbon quantum dots (NS@CQDs) as fluorescent sensors for the assay of the studied drugs. NS@CQDs synthesis was performed via a facile hydrothermal technique using thiosemicarbazide as nitrogen and sulfur source and citric acid as carbon source [33].

Carbon quantum dots (CQDs) have caught researchers' curiosity in different research fields because of their excellent photophysical properties, good solubility, low toxicity, high quantum yield, and eco-friendly nature [34]. Top-down and bottom-up methods are two classical approaches used for the synthesis of CQDs. The top-down methods are used for preparing CQDs by using laser ablation and electrochemical oxidation for breaking larger carbon structures into smaller ones, which produce low yield and require high cost. The bottom-up methods are based on the carbonization of organic elements under thermal, hydrothermal, and solvothermal conditions, which offer additional advantages over top-down methods such as high production yield, and unique photophysical properties, and the processes are economical and easy [35]. The applications of CQDs have increased, including bio-imaging, food industry, drug delivery, light-emitting diodes, photocatalysis, photodetectors, and photodynamic therapy [36].

Doping is the insertion of heteroatoms like (boron, sulfur, nitrogen, or phosphorous) into the general structure of CQDs with or without modification in their surface. Doping of CQDs results in increasing the quantum yield and improving the fluorescence properties of CQDs, which in turn increases their applications [16, 37–39]. NS@CQDs have been repeatedly studied as carbon and nitrogen have the same atomic radius, in addition, sulfur and carbon are actually close in electronegativity [40].

The current study aimed to design a simple spectrofluorimetric method for the assay of the important CNS-acting drugs, OLZ and DZP, in their tablets by studying their quenching effect on the fluorescence of the synthesized NS@CQDs without the need for any pretreatment steps. In addition, the content uniformity testing of the cited drugs was also verified.

## Experimental

### Materials and reagents

Olanzapine (99.0%) and Diazepam (99.20%) were kindly provided by EIPICO (10th of Ramadan City, Egypt). Zyprexa^®^ coated tablets (10 mg OLZ/tablet, Batch No. D423653, a product of Eli Lilly Company), Prexal^®^ (5 mg OLZ/tablet, Batch No.3920824), Olapex^®^ (5 mg OLZ/tablet, Batch No.AT12311220), and Neuril^®^ (5 mg DZP/tablet, Batch No.319206) tablets were bought from a local Pharmacy. Citric acid, thiosemicarbazide, phosphoric acid, potassium dihydrogen phosphate, methanol, and sodium hydroxide were purchased from Sigma-Aldrich (USA).

A human plasma sample was provided by Mansoura University Hospitals (Mansoura, Egypt) and kept frozen until used after gentle thawing. Double distilled water was utilized throughout the study and all chemicals used were of analytical grade. Phosphate buffer (0.02 M) with pH range (3–9.5) was freshly prepared.

### Apparatus

The fluorescence spectra were recorded using CA-95051 Cary Eclipse Fluorescence Spectrophotometer with Xenon light source and software from Agilent Technologies (USA). The excitation and emission monochromators were operated at a voltage of (750 V) with a slit width of 5 nm. The absorption spectra were measured by a double beam spectrophotometer (PG Instrument, UK). Fourier Transform Infrared spectroscopy (FT-IR) spectra were recorded by Thermo Fisher Scientific FT-IR Spectrometer (Nicolet-iS10, USA) across 4000 to 1000 cm^−1^ spectral range and 32 scans at a resolution of 4 cm^−1^. The shape and particle size of the used NS@CQDs were examined by JEM-2100 High-Resolution Transmission Electron Microscopy (HRTEM) (JEOL, Japan). The sample was examined on Cu-grid coated with carbon (200 mesh) using the instrument at (200 kV) voltage. 0.45 µm membrane filters (Phenomenex, USA), ultrasonic bath sonicator (SS-101H-230 model, USA), pH meter (Jenway-3510 model, UK), cooling centrifuge (2-16P model, Germany), and vortex mixer (IVM-300 p model, Taiwan) were also utilized.

### Standard solutions

Standard stock solutions of OLZ and DZP (1.0 mM) were prepared by dissolving each drug in methanol. Accurately measured known concentrations (5.0–200.0 μM) and (1.0–100.0 μM) of OLZ and DZP, respectively, were prepared by diluting the standard solutions using double distilled water. The solutions showed stability for at least 10 days under refrigeration.

### Synthesis of fluorescent NS@CQDs

NS@CQDs were prepared by adopting a facile and rapid hydrothermal technique, which was previously described in the literature [33]. The detailed procedure is as follows: thiosemicarbazide (0.68 g) and citric acid (0.52 g) were mixed with double distilled water (20 mL). The obtained mixture was sonicated for 30 min, then refluxed at 160 °C till the formation of dark orange color. The prepared NS@CQDs solution was then kept in the refrigerator for future use.

### Quantum yield measurement

The NS@CQDs quantum yield (Φ_NS@CQDs_) was obtained by adopting the single point method using the following equation [41, 42]:

1 $$\Phi_{NS@CQDs} = \Phi_{QS} \times \left( {F_{NS@CQDs} /F_{QS} } \right) \, \times \, \left( {A_{QS} /A_{NS@CQDs} } \right) \times \left( {\eta_{NS@CQDs} /\eta_{QS} } \right)^{2}$$

where: Φ denotes the fluorescence quantum yield, F represents the integrated emission intensity, A represents the value of absorbance, η represents solvent refractive index (double distilled water).

Quinine sulphate (QS) was employed as the standard fluorophore. Its quantum yield in 0.1 M H_2_SO_4_ at λ_ex_ = 350 nm is 0.54. In an aqueous solution, η_NS@CQDs_/η_QS_ equals to 1.

### General procedures

#### Construction of calibration curves

Aliquots of stock solutions in the concentration range of 5.0–200.0 μM for OLZ and 1.0–100.0 μM for DZP were transferred into a set of 10 mL volumetric flasks. For DZP, 100.0 μL NS@CQDs were added, and then the flasks were completed to the mark with double distilled water. For OLZ, 100.0 μL of NS@CQDs were added, followed by 2 mL of phosphate buffer of pH 3.5. After that, the flasks were completed with double distilled water and then heated at 50 °C in a water bath for 10 min. The fluorescence intensities of the solutions were measured at 430 nm using 360 nm as an excitation wavelength. The resultant fluorescence quenching spectra were recorded with a blank experiment conducted simultaneously. The decrease in fluorescence intensity (ΔF = F_0_ − F) was plotted versus the drug concentrations (μM) to obtain the calibration curves. Meanwhile, the corresponding regression equations were obtained.

#### Analysis of tablets

Ten tablets of each of Zyprexa^®^, Prexal^®^, and Neuril^®^ preparations were separately weighed and finely pulverized. Then, a quantity of powder equivalent to 10 mg (Zyprexa^®^) or 5 mg (Prexal^®^) of OLZ or 5 mg (Neuril^®^) of DZP was transferred to a small flask. 50 mL of methanol were added to the flask, and then sonicated for 15 min. The contents of the conical flask were then filtered into a volumetric flask (100 mL), and completed to the volume with methanol. Increasing aliquots of the filtrate were transferred to 10 mL volumetric flasks, and the procedures under “Construction of calibration curves” section were then followed. The nominal contents of the tablets were determined using the corresponding regression equation.

#### Determination of DZP in spiked human plasma

One milliliter aliquots of human plasma sample were spiked with various aliquots of DZP stock solution (1.0 mM) in a series of 15 mL centrifuge tubes. The spiked samples were vortex mixed for 1 min before being diluted to 5 mL with methanol, and then centrifuged for 15 min at 6000 rpm. One milliliter aliquots were withdrawn from the clear supernatant and filtered by 0.45 μm membrane filters, transferred into 10 mL volumetric flasks, followed by the addition of 100.0 μL of NS@CQDs. The flask contents were diluted to the mark using double distilled water to get three different concentrations of DZP (1.0, 5.0, and 15.0 μM). The decrease in fluorescence intensities (F_0_ − F) was plotted versus DZP concentrations (μM) to get the regression equation, and then DZP percentage recoveries were obtained.

## Results and discussion

In the current study, NS@CQDs were used as fluorescent nanosensors having strong blue fluorescence intensities that could be quantitatively quenched by both OLZ and DZP, providing a rapid, simple, and economic spectrofluorimetric method for their sensitive analysis.

### Characterization of NS@CQDs

**Fig. 2:**
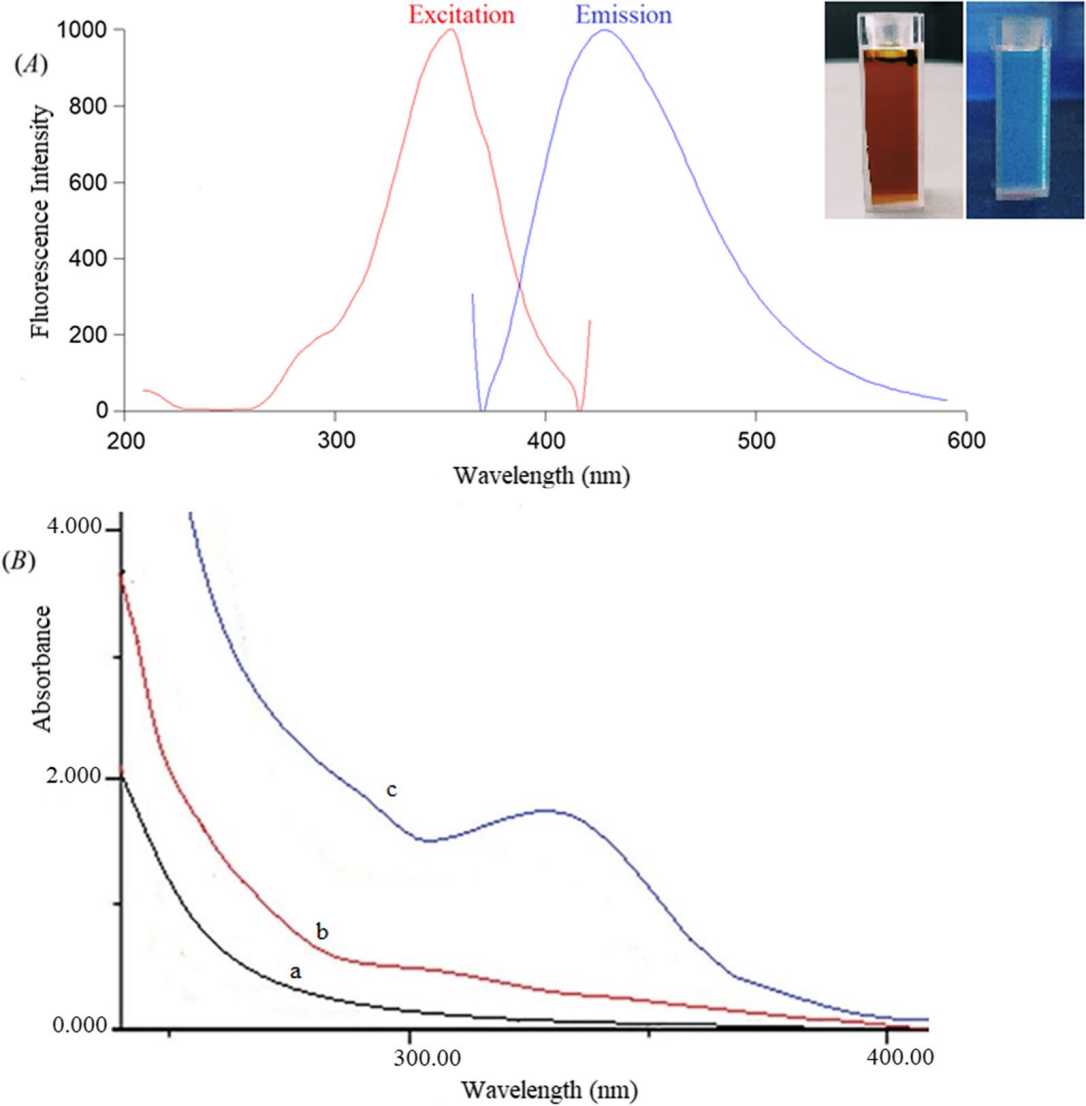
**A** Fluorescence emission spectrum of NS@CQDs at 430 nm after excitation at 360 nm (Inset: photographs of N-CQDs under visible light and UV light), **B** the UV–Vis absorption spectra of (a) citric acid, (b) thiosemicarbazide, (c) NS@CQDs

Fluorescence spectroscopy and UV–Visible absorption spectroscopy were used to characterize the optical characteristics of NS@CQDs. The fluorescence intensity of NS@CQDs was obtained at 430 nm after excitation at 360 nm, as illustrated in Fig. 2A. The prepared NS@CQDs emitted blue luminescence when irradiated under UV light (Fig. 2A), and the solution was stable for at least two weeks when kept at 4 °C without any apparent alteration. The study showed that the excellent hydrophilicity and the NS@CQDs fluorescence are related to N–H, O–H, and C=O groups [40]. The UV–Vis absorption spectra of thiosemicarbazide, citric acid, and NS@CQDs are illustrated in Fig. 2B. As observed, NS@CQDs have a distinct peak around 320 nm, which was caused by the surface states trapping excited-state energy [43, 44]. The UV absorption spectrum of NS@CQDs in the presence of OLZ and DZP is illustrated in Additional file 1: Fig. S1. Also, the synthesized NS@CQDs exhibited a high fluorescence quantum yield of 50%.

**Fig. 3:**
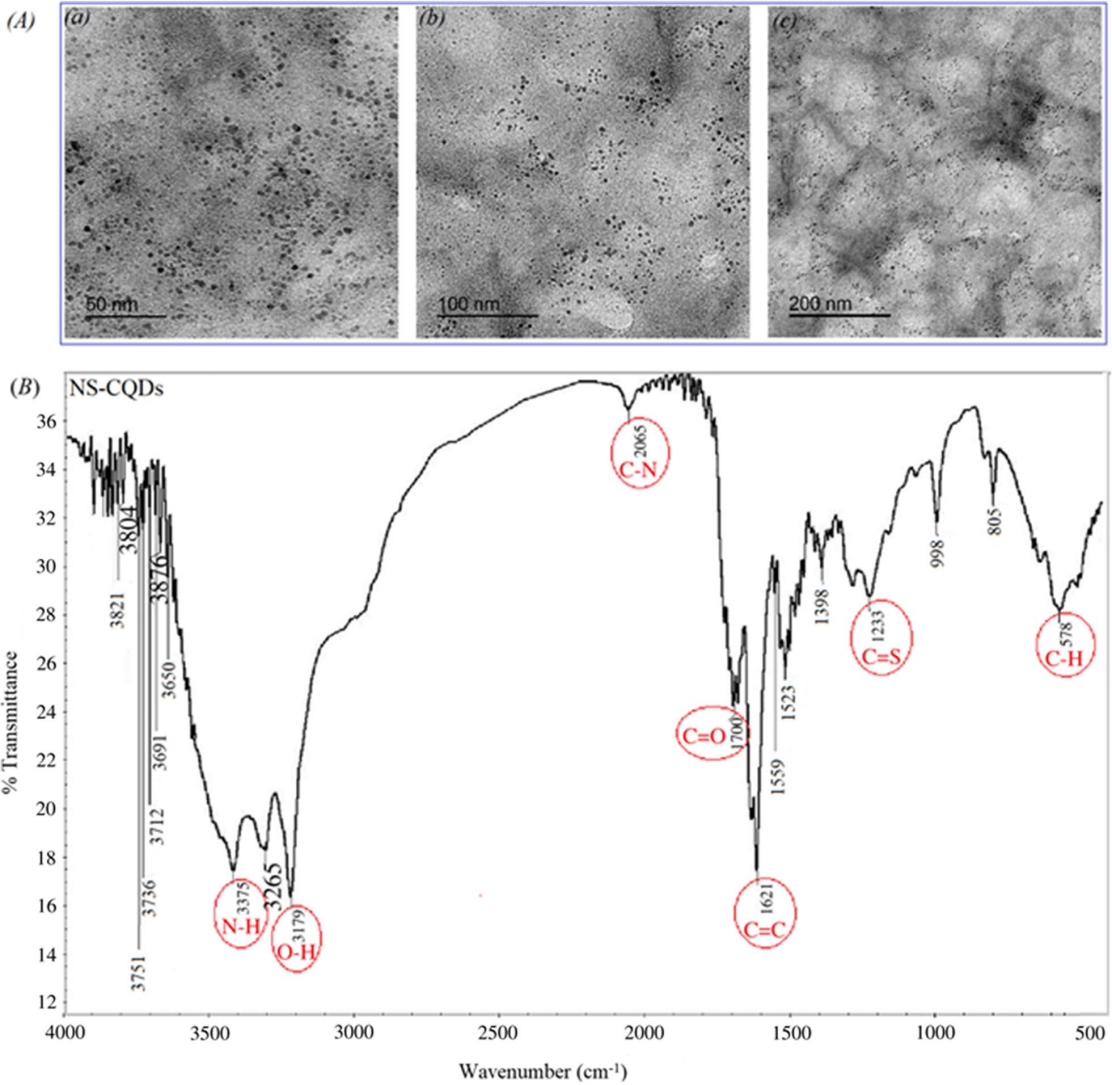
**A** HRTEM images of NS@CQDs (a) 50 nm, (b) 100 nm, (c) 200 nm, **B** FT-IR spectrum of NS@CQDs

HRTEM was also used to examine the shape and size of NS@CQDs. HRTEM images showed that NS@CQDs were well distributed with spherical shapes and separated from each other without obvious aggregation with a particle size in the range of 5–10 nm (Fig. 3A).

Characterization using FT-IR was also performed to identify the surface functional groups of NS@CQDs. As presented in Fig. 3B, N–H/O–H stretching bands are displayed at 3500–3100 cm^−1^. The characteristic stretching peaks at 2065, 1700, 1621, and 1233 cm^−1^ represent C–N, C=O, C=C, and C=S, respectively. The bending peak at 578 cm^−1^ represents C-H [45].

### Fluorescence quenching mechanism

**Fig. 4:**
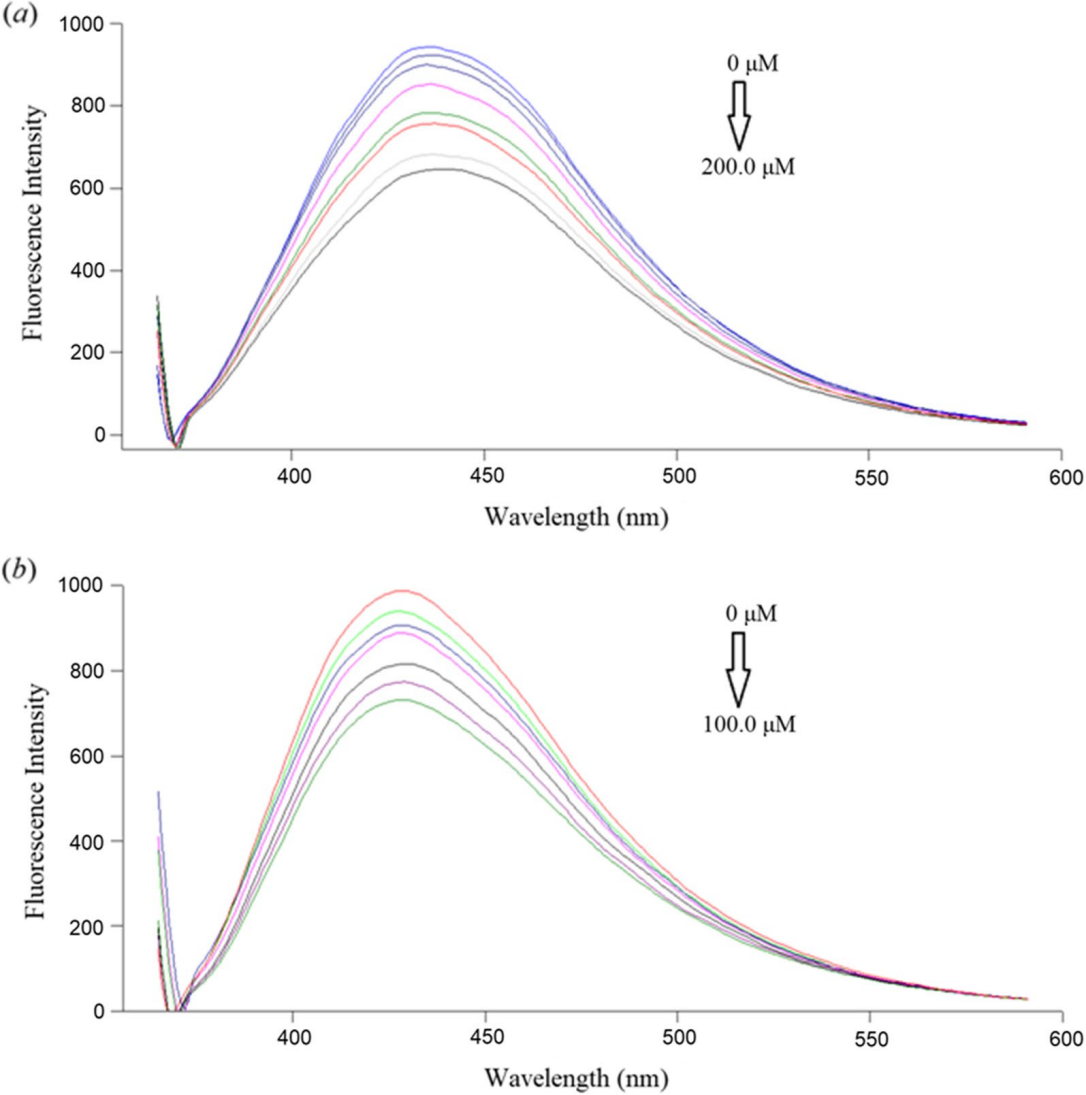
Fluorescence emission spectra of NS@CQDs in the presence of various concentrations of **a** OLZ (from top to bottom: 0, 5.0, 25.0, 50.0, 85.0, 115.0, 185.0, 200.0 μM), **b** DZP (from top to bottom: 0, 1.0, 10.0, 25.0, 50.0, 75.0, 100.0 μM)

The NS@CQDs fluorescence was found to decrease in a quantitative manner with increasing concentrations of each of OLZ and DZP as illustrated in Fig. 4. Generally, the quenching mechanisms could be resolved into different categories, such as dynamic quenching, static quenching, and inner filter effect (IFE). Dynamic quenching can be distinguished from static one by lifetime measurements or preferably by varying dependence on temperature. Higher temperature settings result in faster diffusion and an increase in Stern–Volmer quenching constant (K_SV_) in dynamic quenching [46, 47]. In contrast, in static quenching, higher temperature settings cause complex dissociation and a decrease in K_SV_ [48]. The K_SV_ is unaffected by temperature in the case of IFE [49].

In this study, UV absorption spectra of each of OLZ and DZP overlapped with the excitation spectra of NS@CQDs (Additional file 1: Fig. S2), which indicates the possibility of IFE. The correction of fluorescence intensity of NS@CQDs for possible IFE was studied with increasing concentrations of each of OLZ and DZP using the following equation [49]:

2 $$F_{corr} = \, F_{obs} \times 10^{{\left( {A_{ex} + A_{em} } \right)/2}}$$

where: F_corr_ and F_obs_ represent the corrected and observed fluorescence intensities, A_em_ and A_ex_ are the sum of the absorbance values of the drug at the emission and excitation wavelengths of NS@CQDs, respectively.

For both corrected and observed fluorescence intensities, the suppressed efficiency (% E) were obtained using the following equation:

3 $$\% E \, = \, \left[ {1 - \left( {F/F_{0} } \right)} \right] \times 100$$

**Fig. 5:**
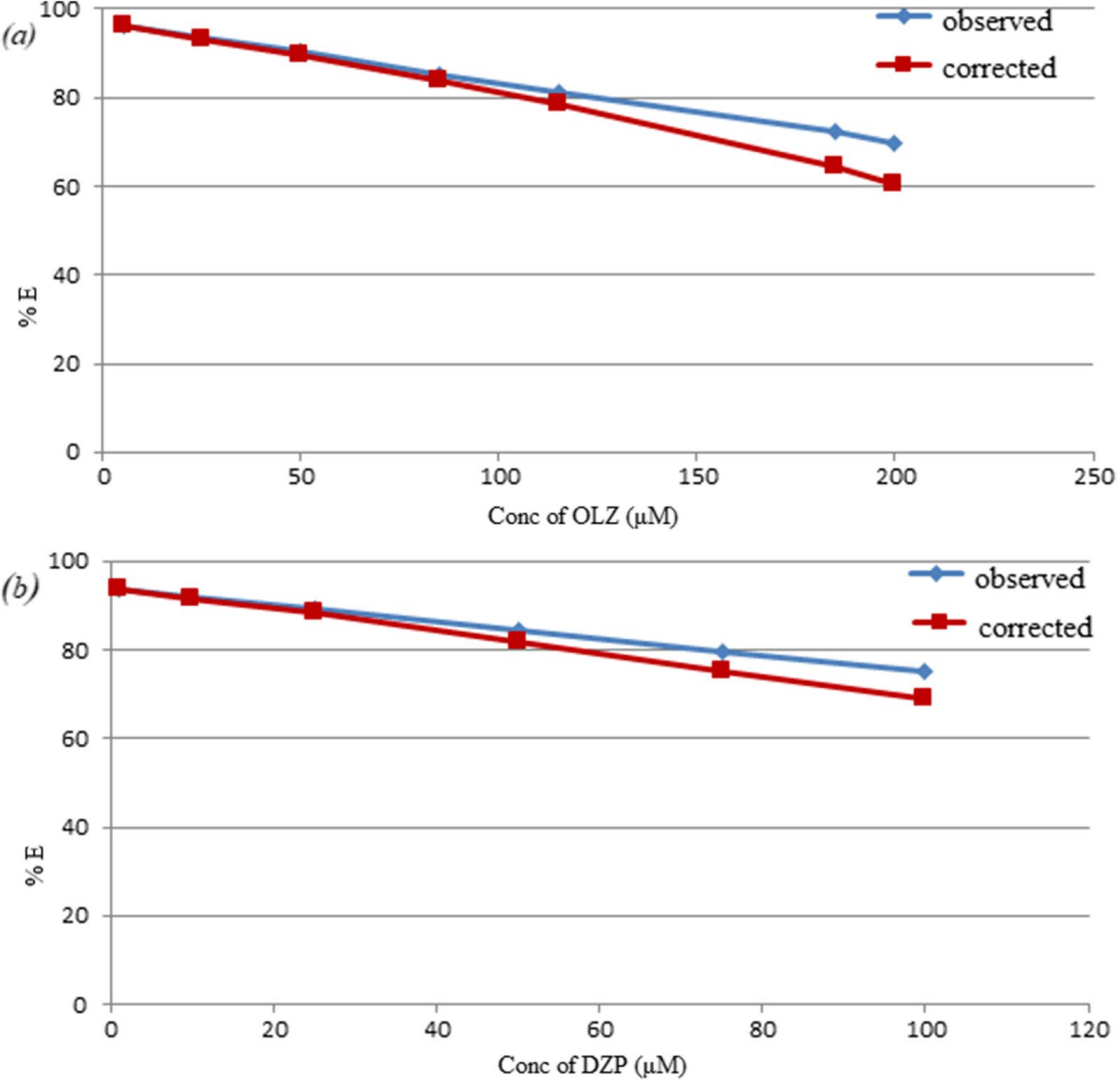
The suppressed efficiency (% E) of corrected and observed fluorescence of NS@CQDs in presence of different concentrations of **a** OLZ (5.0, 25.0, 50.0, 85.0, 115.0, 185.0, 200.0 μM), **b** DZP (1.0, 10.0, 25.0, 50.0, 75.0, 100.0 μM)

The % E values of both corrected and observed fluorescence intensities of NS@CQDs were plotted against drug concentrations. Figure 5 revealed that IFE has a weak effect on the quenching of the fluorescence of NS@CQDs by the studied drugs.

The Stern–Volmer relationship was used to determine the possible quenching mechanism:

4 $$F_{0} /F \, = \, 1 \, + \, K_{sv} \left[ Q \right] \, = \, 1 \, + \, K_{q} \tau_{0} \left[ Q \right]$$

where: F_0_ and F denote the measured fluorescence intensities in the absence and presence of a quencher (OLZ or DZP), respectively, K_sv_ denotes the Stern–Volmer quenching constant, k_q_ refers to the bimolecular quenching rate coefficient, τ_0_ refers to the average fluorophore lifetime (10^–8^ s), [Q] refers to the quencher concentration (OLZ or DZP) [50].

**Fig. 6:**
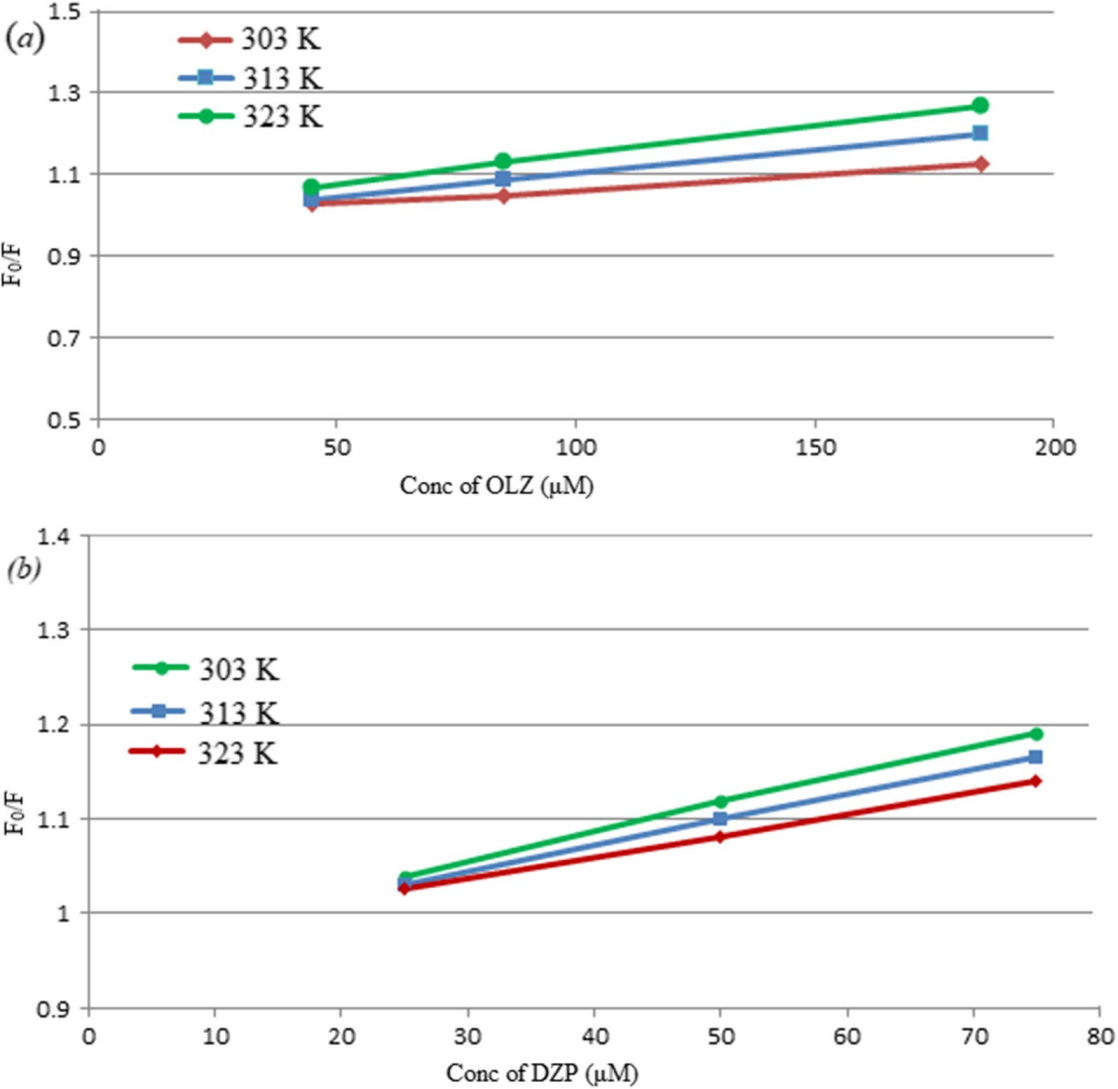
Stern–Volmer plots for the quenching of the fluorescence of NS@CQDs at three different temperature settings (303, 313, and 323 K) by different concentrations of **a** OLZ (45.0, 85.0, 185.0 μM), **b** DZP (25.0, 50.0, 75.0 μM)

The quenching experiments were performed at three temperature settings (303, 313, 323 K). For OLZ, K_sv_ values calculated by the previous equation were found to be 0.671 × 10^3^, 1.094 × 10^3^, and 1.45 × 10^3^ L/mol at 303, 313, and 323 K, respectively (Fig. 6a). It is evident that K_sv_ increased as the temperature increased; therefore, the fluorescence quenching mechanism of NS@CQDs by OLZ was concluded to be dynamic rather than static. While for DZP, the K_sv_ values were found to be 2.60 × 10^3^, 2.26 × 10^3^, and 1.90 × 10^3^ L/mol at 303, 313, and 323 K, respectively (Fig. 6b), indicating that the fluorescent sensing mechanism with DZP was related to static quenching mechanism as K_sv_ values decrease with a rise in temperature [48].

### Experimental conditions optimization

#### Effect of pH

The impact of pH on the fluorescence quenching of NS@CQDs by each of OLZ or DZP was studied using phosphate buffer within the pH range of (3–9.5). It was found that phosphate buffer with pH 3.5 caused a subsequent increase in the quenching of NS@CQDs fluorescence by OLZ (Additional file 1: Fig. S3a). Accordingly, the influence of phosphate buffer volume (pH 3.5) was also determined using various volumes of buffer within the range of (0.5–3.0 mL), and 2.0 mL was found to be the optimum volume (Additional file 1: Fig. S3b). On the other hand, changing pH values had a negligible impact on the quenching of NS@CQDs fluorescence by DZP, so buffer was excluded in further studies with DZP (Additional file 1: Fig. S3a).

#### Effect of incubation time

The time impact on quenching of the NS@CQDs fluorescence by the investigated drugs was also studied at 5-min time intervals from 1 to 30 min. The maximum quenching of NS@CQDs fluorescence with OLZ was obtained after 10 min, while accomplished within about 1 min with DZP (Additional file 1: Fig. S4a).

#### Effect of temperature

The temperature influence on the fluorescence quenching was studied after the addition of either OLZ or DZP to the synthesized NS@CQDs over the range of 25–60 °C. The fluorescence quenching with OLZ increased with increasing temperature, up to 50 °C, then it remained almost constant, so 50 °C was used as the optimum temperature in a water bath for 10 min as the optimum incubation time (Additional file 1: Fig. S4b), while increasing the temperature caused the fluorescence quenching to decrease with DZP, so its study was carried out at room temperature.

### Method validation

The proposed method was validated in compliance with ICHQ2 (R1) recommendations [51].

#### Linearity and range

The plots of fluorescence quenching (F_0_ − F) versus drug concentrations were rectilinear within the range of (5.0–200.0 μM) and (1.0–100.0 μM) for OLZ and DZP, respectively. The following equations can be used to express linear regression data analysis:

5 $$\left( {F_{0} - F} \right) \, = \, 0.62 \, C \, + \, 11.70 \;\; \left( {r \, = \, 0.9995} \right) \;for\;OLZ$$

6 $$\left( {F_{0} - F} \right) \, = \, 1.41 \, C \, + \, 44.95 \;\; \left( {r \, = \, 0.9998} \right) \;\;for\;\;DZP$$

where: F_0_ and F denote the measured fluorescence intensities in the absence and presence of the studied drugs (OLZ and DZP), respectively, C denotes OLZ or DZP concentration (μM), and r is the correlation coefficient.

The linear regression data analysis for the proposed method was illustrated in Table 1.

**Table 1:** Analytical performance data for OLZ and DZP determination using the suggested method

Parameters	OLZ	DZP
Concentration range (µM)	5.0–200.0	1.0–100.0
LOD (µM)	0.68	0.29
LOQ (µM)	2.07	0.89
Regression equation	(F_0_ − F) = 0.62C + 11.70	(F_0_ − F) = 1.41C + 44.95
Correlation coefficient (r)	0.9995	0.9998
S.D. of the intercept (S_a_)	0.13	0.12
S.D. of the slope (S_b_)	0.001	0.002
S.D. of the residuals (S_y/x_)	0.20	0.19
Percentage relative standard deviation (% RSD)	1.669	1.022
Percentage relative error (% error)	0.62	0.42

#### Limit of detection (LOD) and limit of quantitation (LOQ)

LOD and LOQ values were obtained using the following equations [52]: LOD = 3.3 S_a_/b, LOQ = 10 S_a_/b, Where S_a_ denotes the standard deviation of the intercept of the regression line and b denotes its slope. The obtained results showed that the adopted method could be used for the assay of the investigated drugs with acceptable sensitivity (Table 1).

#### Accuracy and precision

The results obtained by the suggested method were examined and compared with those obtained by comparison methods [53, 54]. The results were statistically analyzed by adopting the Student's t-test and Variance ratio F-test [52], which proved that there were insignificant differences between the methods regarding accuracy and precision, respectively (Table 2). In addition, intra-day and inter-day precisions were studied, and small % RSD values (< 1.57) and low % error values (< 0.91) were obtained, pointing out the acceptable precision of the current method (Additional file 1: Table S1).

**Table 2:** Results of determination of the cited drugs in raw materials by the suggested method

Parameter	OLZ			DZP		
	Conc. taken (μM)	Conc. found (μM)	% recovery^a^	Conc. taken (μM)	Conc. found (μM)	% recovery^a^
	5.0	4.85	97.12	1.0	0.98	98.23
	25.0	24.65	98.60	10.0	9.80	98.00
	50.0	49.58	99.16	25.0	24.96	99.84
	85.0	87.17	102.56	50.0	50.22	100.45
	115.0	114.01	99.15	75.0	74.85	99.80
	185.0	184.45	99.70	100.0	100.01	100.01
	200.0	200.30	100.14			
Mean			99.49			99.39
± SD			1.66			1.02
% RSD			1.669			1.022
% error			0.62			0.42

	Comparison method [53]	Comparison method [54]				
Mean ± SD	100.30 ± 1.60	98.45 ± 1.02				
N^c^	3.0	3.0				
t-value	0.72 (2.31)^b^	1.31 (2.36)^b^				
F-value	1.07 (19.32)^b^	1.02 (19.30)^b^				

^a^Average of three separate determinations

^b^Values in parenthesis are the tabulated values of t and F at p = 0.05 [52]

^c^Number of samples

#### Robustness

The robustness of the developed method was investigated by studying the impact of slight changes in the experimental conditions influencing the quenching of NS@CQDs fluorescence by the studied drugs. For OLZ, these conditions include: NS@CQDs volume (100.0 μL ± 5), incubation time (10 min ± 2 min), phosphate buffer pH (3.5 ± 0.2), volume of phosphate buffer (2.0 mL ± 0.2 mL), and temperature (50 °C ± 2 °C). While for DZP, these conditions include: NS@CQDs volume (100.0 μL ± 5) and incubation time (1 min ± 0.5 min). The obtained results demonstrated that small changes in experimental parameters did not exhibit any significant effects on the performance of the developed method, as presented in Additional file 1: Table S2.

#### Selectivity

The adopted method was effectively applied for the assay of both drugs in tablets with high % recoveries and low % RSD values (< 2%) without interference from the common tablet excipients, indicating that the current method is highly selective (Table 3). The method selectivity was also achieved by its ability to detect OLZ in the presence of other CNS-acting drugs, such as fluoxetine and risperidone, without interference. The experimentally determined tolerance limits were 200.0 μM for fluoxetine and 50.0 μM for risperidone. The tolerance limits were calculated as the concentration that results in a 2% relative error [55]. Moreover, the current method could be efficiently used to analyze DZP in plasma samples with acceptable % recoveries and low % RSD values.

**Table 3:** Results of OLZ and DZP determination in tablets using the suggested method

Preparation	Proposed method			Comparison method [53]
	Conc. taken (μM)	Conc. found (μM)	% recovery^a^	% recovery^a^
Zyprexa^®^ coated tablets (OLZ, 10 mg/tablet)	16.0	16.24	101.50	99.80
	32.0	31.62	98.84	101.10
	48.0	48.56	101.17	101.50
Mean			100.50	100.80
± SD			1.45	0.89
% RSD			1.443	0.882
t-value	0.30 (2.77)^b^			
F-value	2.66 (19.00)^b^			
Prexal^®^ tablets (OLZ, 5 mg/tablet)	16.0	16.07	100.45	98.33
	32.0	31.84	99.50	99.25
	48.0	47.81	99.61	97.40
Mean			99.85	98.33
± SD			0.52	0.93
% RSD			0.521	0.941
t-value	2.49 (2.77)^b^			
F-value	3.16 (19.00)^b^			

Preparation	Proposed method			Comparison method [54]
	Conc. taken (μM)	Conc. found (μM)	% recovery^a^	% recovery^a^
Neuril^®^ tablets (DZP, 5 mg/tablet)	15.0	15.16	101.06	100.90
	25.0	25.43	101.70	102.34
	35.0	35.18	100.50	100.80
Mean			101.09	101.35
± SD			0.60	0.86
% RSD			0.594	0.851
t-value	0.43 (2.77)^b^			
F-value	2.06 (19.00)^b^			

^a^Average of three separate determinations

^b^Values in parenthesis are the tabulated values of t and F at p = 0.05 [52]

### Method applications

#### Analysis of OLZ and DZP in tablets

The current method was employed for the quantitative analysis of each of OLZ and DZP in their tablets. Based on the corresponding regression equations, the drug concentrations in tablets were determined. The average % recoveries of drug concentrations in various tablet formulations were acceptable as demonstrated in Table 3. The results of the suggested method showed no significant differences with those obtained with comparison methods [53, 54], demonstrating that the proposed method has good precision and accuracy.

#### Content uniformity testing of the tablets

Because of the high sensitivity and selectivity of the current method, it was possible to determine the drugs concentrations in their low-dose tablets. Ten tablets of each drug were analyzed individually using the same procedures mentioned in “Analysis of tablets” section. The official USP guidelines (The United States Pharmacopeia 36 NF 31) were used to evaluate the uniformity of tablets content [29]. The acceptance value (AV) calculated was less than the maximum allowed acceptance value (L1) stated by USP [30], as presented in Table 4.

**Table 4:** Results of content uniformity testing of OLZ and DZP tablets by the suggested method

Parameter	Tablet no.	% of the labeled claim		
		OLZ		DZP
		Prexal^®^ (5 mg/tablet)	Olapex^®^ (5 mg/tablet)	Neuril^®^ (5 mg/tablet)
		% recovery	% recovery	% recovery
	1	101.40	102.90	100.98
	2	100.15	99.80	100.00
	3	103.90	98.50	101.74
	4	100.00	100.26	103.30
	5	100.98	100.14	99.70
	6	100.90	101.86	102.46
	7	103.70	97.09	101.40
	8	101.00	98.46	101.00
	9	98.00	101.95	102.74
	10	98.19	101.77	97.50
Mean		100.82	100.27	101.08
S.D		1.95	1.86	1.70
% RSD		1.933	1.859	1.683
% error		0.62	0.59	0.76
Acceptance value (AV)		4.68	4.47	4.08
Maximum allowed AV (L1)		15.0	15.0	15.0

#### Analysis of DZP in spiked human plasma

The developed method could be effectively used to analyze DZP in spiked plasma samples. The reported maximum plasma concentration (C_max_) of DZP is about 0.49–0.67 μM after a single oral dose of 10 mg [56]. Although the sensitivity of the proposed method did not reach the limit of C_max_ of the drug, the method's ability to detect the drug in the plasma matrix can be appropriate in the case of drug abuse, taking into consideration that DZP is one of the most commonly abused benzodiazepines. The drug concentration measurements in spiked human plasma were estimated as illustrated in “Determination of DZP in spiked human plasma” section. Using the developed method, a rectilinear relationship was established by plotting the fluorescence quenching (F_0_ − F) versus the DZP concentration in μM. The mean % recoveries ± SD of DZP in spiked plasma were 98.96 ± 0.99 (Table 5). The following equation can be used to express linear regression data analysis:

7 $$\left( {F_{0} - F} \right) \, = \, 9.42 \, C \, + \, 74.95 \;\; \left( {r \, = \, 0.9998} \right)$$

**Table 5:** Results of determination DZP in spiked human plasma by the suggested method

Parameter	Conc. taken (μM)	Conc. found (μM)	% recovery^a^
	1.0	0.98	98.92
	5.0	4.90	98.00
	15.0	14.99	99.97
Mean	98.96		
± SD	0.99		
% RSD	0.996		

^a^Average of three separate determinations

## Conclusion

The developed work introduces a facile, eco-friendly, and economical spectrofluorimetric method for assaying each OLZ and DZP in their tablets. The developed method depends on using NS@CQDs as fluorescent probes for assay of the cited drugs. Thiosemicarbazide and citric acid are the two precursors used for hydrothermal preparation of highly water soluble NS@CQDs. The synthesized NS@CQDs were characterized by different spectroscopic and microscopic techniques. Increasing concentrations of each OLZ and DZP quantitatively quenched the fluorescence of the prepared NS@CQDs, providing a simple spectrofluorometric approach for their sensitive analysis. The mechanism of quenching was found to be dynamic quenching with OLZ and static quenching with DZP. The developed method could be used to analyze the investigated drugs in pure form and tablets with high % recoveries (98.84–101.70%) and low % RSD values (< 2%). The current approach can be applied to detect DZP in the spiked plasma with acceptable % recoveries (98.00–99.97%) and the method's significance is magnified in case of its potential abuse. In addition, the content uniformity testing of the cited drugs was also verified.

## Supplementary Information

**Additional file 1.** Additional figures and tables.

## Data Availability

The datasets generated and/or analyzed during the current study are available from the corresponding author on reasonable request.
