# Supplementary material for: Nitrogen and sulfur-doped carbon quantum dots as fluorescent nanoprobes for spectrofluorimetric determination of olanzapine and diazepam in biological fluids and dosage forms: application to content uniformity testing
Source: BMC Chem. 2022 Nov 15;16(1):98. doi: 10.1186/s13065-022-00894-y (PMC9667599; doi:10.1186/s13065-022-00894-y)
Supplement: Supplementary file 1 — Additional file 1. Additional figures and tables. [file 13065_2022_894_MOESM1_ESM.pdf]

Supplementary Information

Nitrogen and sulfur-doped carbon quantum dots as fluorescent nanoprobes for spectrofluorimetric determination of olanzapine and diazepam in biological fluids and dosage forms: Application to content uniformity testing

Galal Magdy[1*], Noura Said[1], Ramadan A. El-Domany[2], Fathalla Belal[3]

[1] Pharmaceutical Analytical Chemistry Department, Faculty of Pharmacy, Kafrelsheikh University, Kafrelsheikh, P.O. Box 33511, Egypt.

[2] Microbiology and Immunology Department, Faculty of Pharmacy, Kafrelsheikh University, Kafrelsheikh, P.O. Box 33511, Egypt.

[3] Pharmaceutical Analytical Chemistry Department, Faculty of Pharmacy, Mansoura University, Mansoura, P.O. Box 35516, Egypt.

***Corresponding author:** Galal Magdy

E-mail address: galal_magdy@pharm.kfs.edu.eg

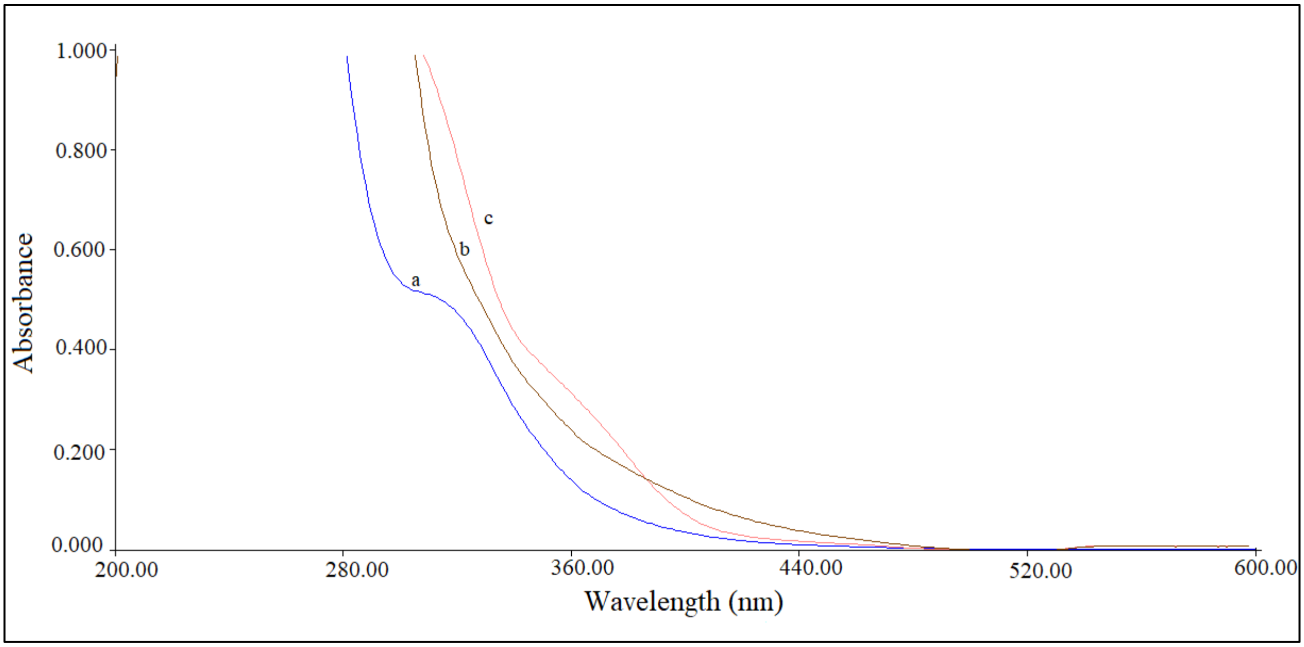

Figure S1 UV-Vis absorption spectra of (a) NS@CQDs, (b) a mixture of NS@CQDs with 100.0 μM OLZ, (c) a mixture of NS@CQDs with 100.0 μM DZP.

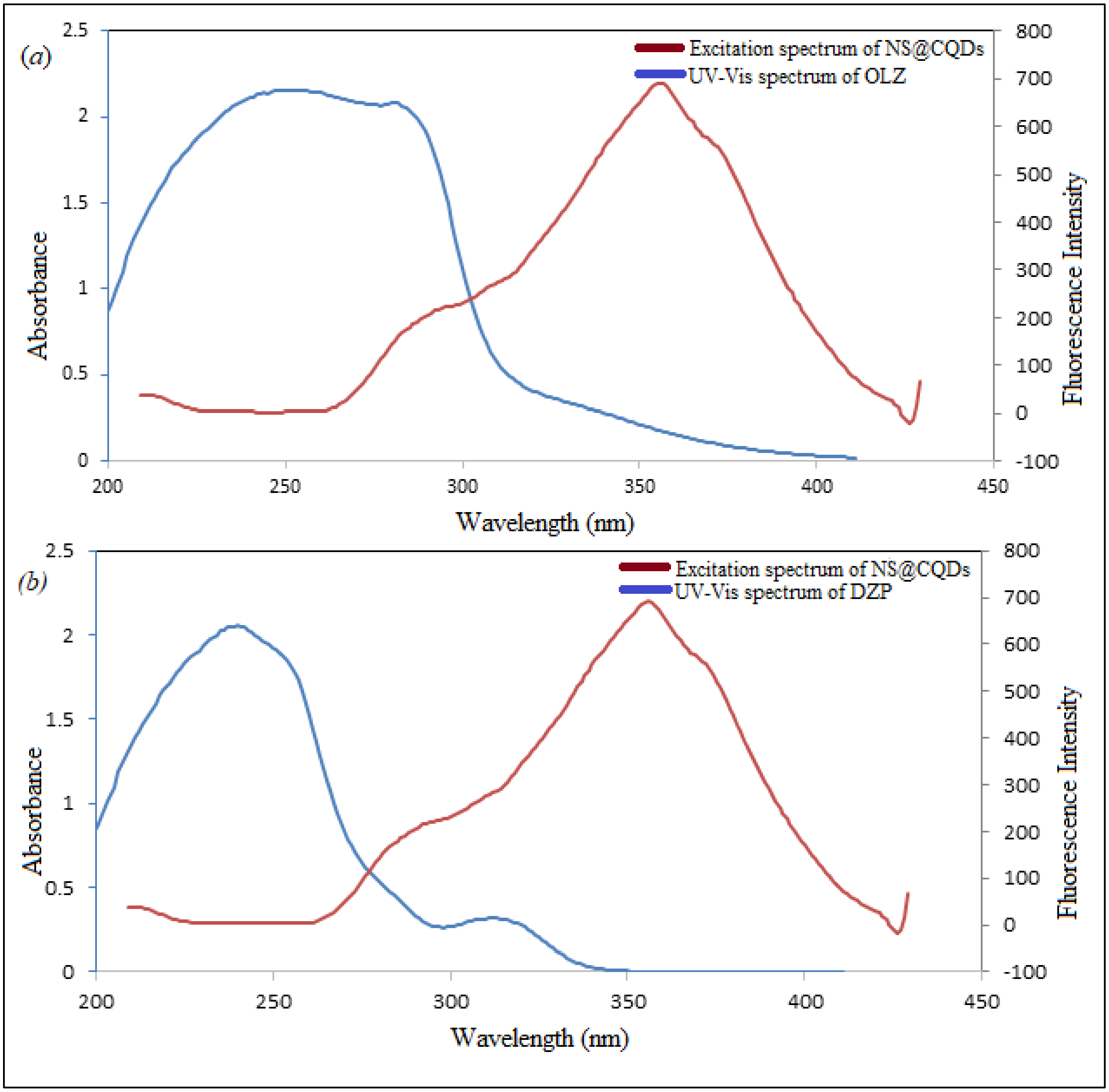

Figure S2 A Co-plot of the excitation spectrum of the NS@CQDs and (a) the absorption spectrum of OLZ (200.0 μM), (b) the absorption spectrum of DZP (100.0 μM).

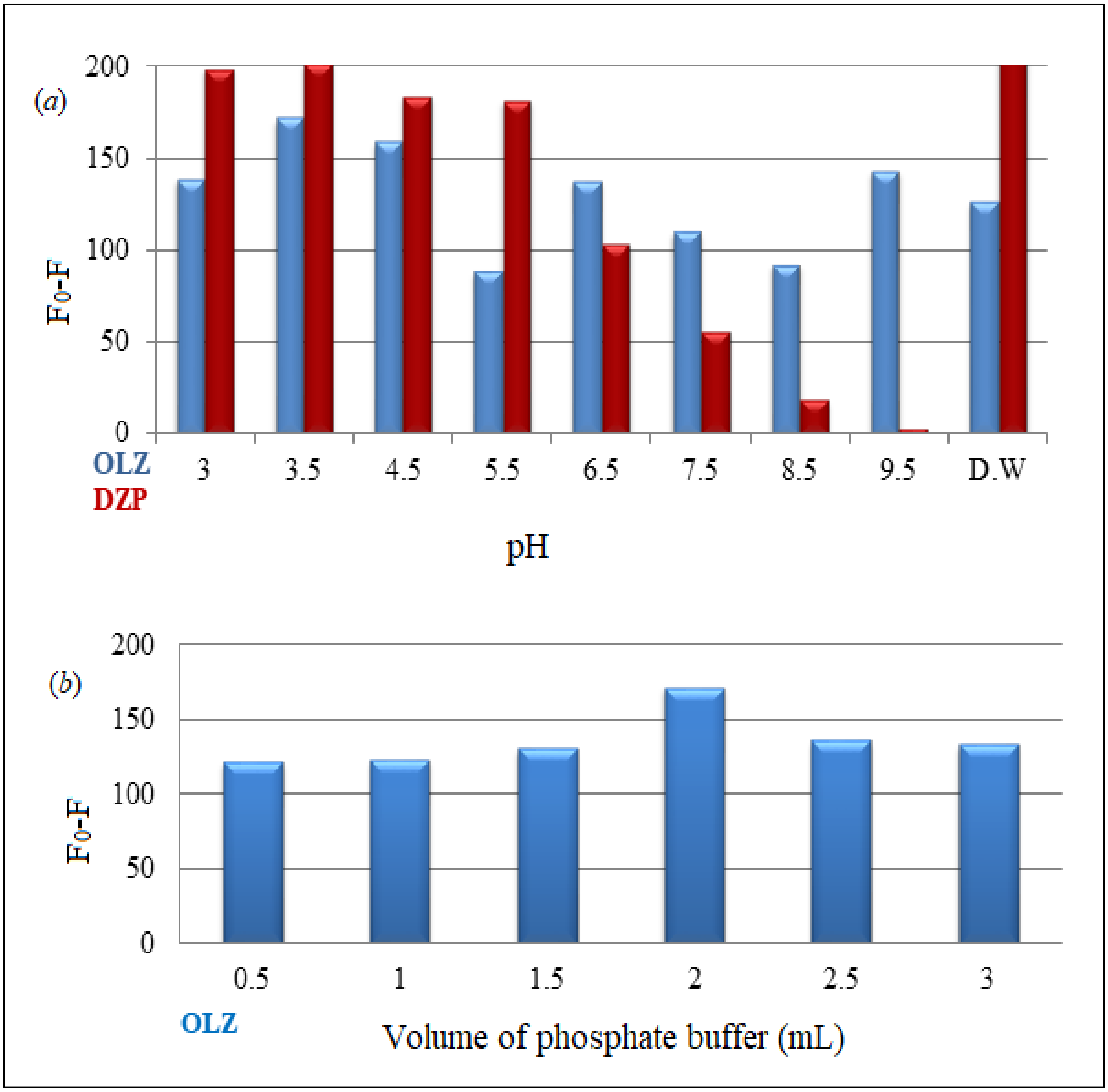

Figure S3 (a) Effect of pH on fluorescence quenching of NS@CQDs by OLZ (100.0 μM) and DZP (100.0 μM), (b) Effect of volume of phosphate buffer (pH 3.5) on fluorescence quenching of NS@CQDs by OLZ (100.0 μM).

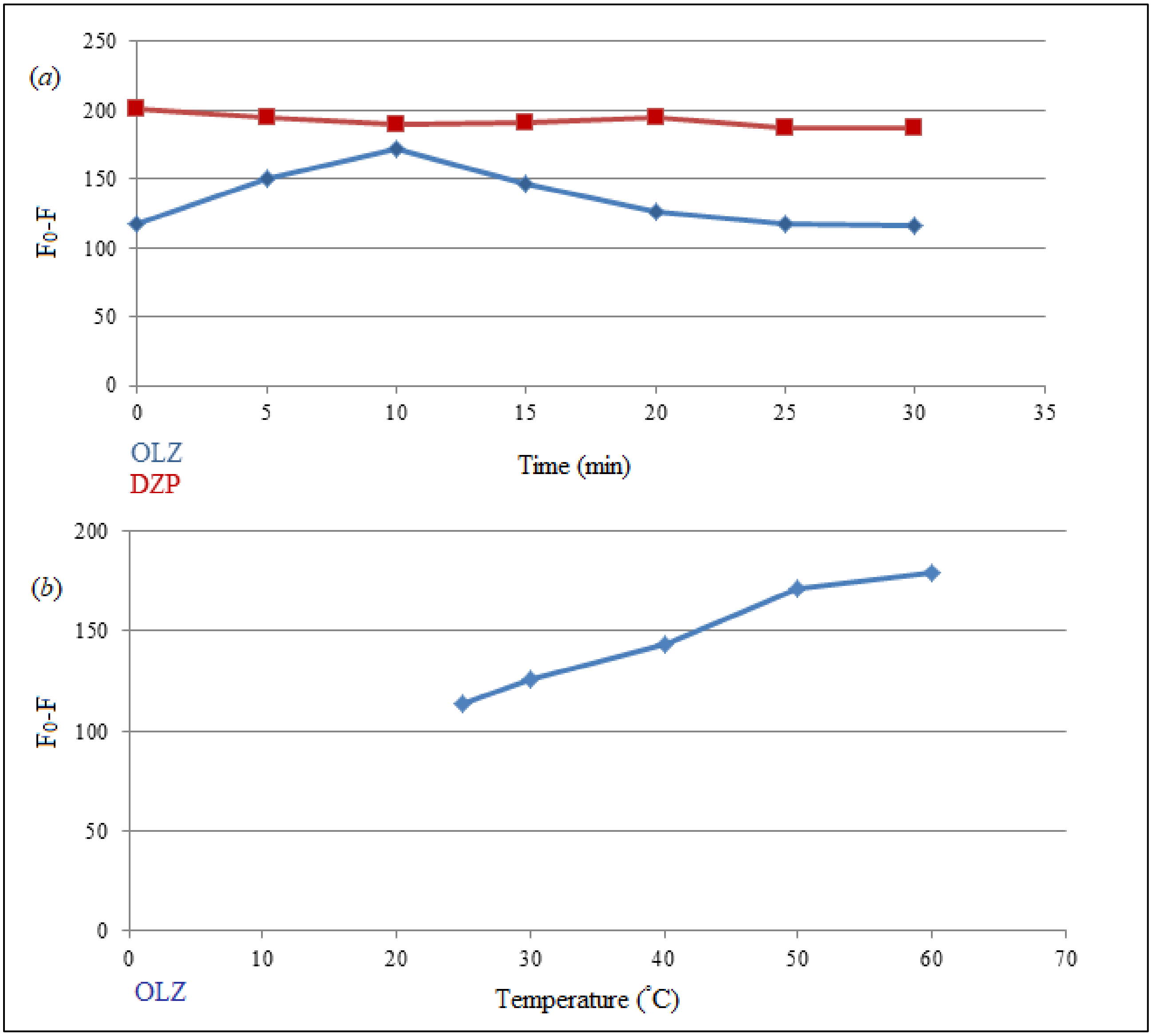

Figure S4 (a) Effect of incubation time on fluorescence quenching of NS@CQDs by OLZ (100.0 µM) and DZP (100.0 µM)), (b) Effect of temperature on fluorescence quenching of NS@CQDs by OLZ (100.0 µM).

Table S1: Intra-day and inter-day precision data for the determination of the cited drugs by the proposed method.

Analyte	Conc. taken (µM)	Intra-day[a]			Inter-day[b]		
		Conc. found (µM) ± SD	% RSD	% error[c]	Conc. found (µM) ± SD	% RSD	% error[c]
**OLZ**	**25.0**	24.87±0.76	0.76	0.44	24.92±0.44	0.44	0.26
	**85.0**	84.91±0.56	0.56	0.32	85.01±0.94	0.94	0.54
	**185.0**	185.34±0.89	0.89	0.51	185.12±0.64	0.64	0.37
**DZP**	**25.0**	24.97±0.75	0.75	0.43	25.09±1.12	1.12	0.64
	**50.0**	50.23±0.45	0.45	0.26	50.03±0.55	0.55	0.32
	**75.0**	75.39±1.57	1.57	0.91	75.31±0.81	0.81	0.47

Each result is the average of three separate determinations.

[a] Within day.

[b] Three successive days.

[c] % error = % RSD/ $\sqrt{n}$.

Table S2: Robustness evaluation of the proposed method.

Factor	OLZ	
**1- NS@CQDs volume (100.0 µL ± 5)**	**% Recovery**	**%RSD**
**95.0 µL**	100.36	0.94
**100.0 µL**	100.72	0.88
**105.0 µL**	100.52	0.99
**2- Incubation time (10 min ± 2 min)**	**% Recovery**	**%RSD**
**8 min**	100.60	0.97
**10min**	100.72	0.88
**12min**	100.40	0.98
**3- Phosphate buffer pH (3.5 ± 0.2)**	**% Recovery**	**%RSD**
**3.3**	100.05	1.02
**3.5**	100.72	0.88
**3.7**	100.38	1.02
**4- Volume of phosphate buffer (2.0 mL ± 0.2 mL)**	**% Recovery**	**%RSD**
**1.8 mL**	100.53	1.02
**2.0 mL**	100.72	0.88
**2.2 mL**	100.39	1.09
**5- Temperature (50°C ± 2°C)**	**% Recovery**	**%RSD**
**48°C**	100.55	1.03
**50°C**	100.72	0.88
**52°C**	100.43	1.09
**Factor**	**DZP**	
**1- NS@CQDs volume (100.0 µL ± 5)**	**% Recovery**	**%RSD**

**95.0 µL**	100.10	0.97
**100.0 µL**	99.66	0.93
**105.0 µL**	100.17	1.08
**2- Incubation time (1min ± 0.5 min)**	**% Recovery**	**%RSD**
**0.5 min**	100.25	1.09
**1 min**	99.66	0.93
**1.5 min**	100.28	1.04
